# Real-World Evaluation of the HELPP Score and CALLY Index for Preoperative Prognostic Stratification in Resectable Pancreatic Ductal Adenocarcinoma

**DOI:** 10.3390/jcm15010312

**Published:** 2025-12-31

**Authors:** İlkay Çıtakkul, Umut Kefeli, Khatira Shukurova, Zehra Aytin, Yasemin Bakkal Temi, Ece Baydar, Kazım Uygun, Devrim Çabuk

**Affiliations:** Department of Internal Medicine and Medical Oncology, Kocaeli University, 41380 Kocaeli, Turkey; ukefeli@yahoo.com (U.K.); shukurova.khatira@mail.ru (K.S.); dr.zehraonesu@gmail.com (Z.A.); yasemin.temi@kocaeli.edu.tr (Y.B.T.); ecebaydar@gmail.com (E.B.); kzuygun@hotmail.com (K.U.); devrimcabuk@yahoo.com (D.Ç.)

**Keywords:** pancreatic ductal adenocarcinoma, HELPP score, CALLY index, prognosis, survival

## Abstract

**Background/Objectives**: Preoperative prognostic assessment is essential for optimizing treatment strategies in pancreatic ductal adenocarcinoma (PDAC). This study aimed to evaluate and compare the prognostic value of the Heidelberg Pancreatic Prognostic (HELPP) score and the C-reactive protein–albumin–lymphocyte (CALLY) index in patients with resectable PDAC. **Methods**: We retrospectively analyzed clinical and laboratory data of 109 patients with resectable PDAC who underwent curative-intent surgery and adjuvant therapy. Patients were stratified based on preoperative HELPP and CALLY scores. Overall survival (OS) and disease-free survival (DFS) were assessed using Kaplan–Meier analysis, while independent prognostic factors were determined through multivariate Cox regression. **Results**: Kaplan–Meier survival analyses demonstrated that a HELPP score > 3 and a low CALLY index (≤1.029) were significantly associated with worse OS and DFS (log-rank *p* < 0.05). In multivariate analysis, the HELPP score was identified as an independent predictor of survival, whereas the CALLY index, although associated with survival in univariate analysis, did not reach statistical significance. In ROC analysis, both models exhibited acceptable discrimination, with the HELPP score achieving superior AUC values in predicting 1-year OS compared to the CALLY index. **Conclusions**: The HELPP score demonstrated independent prognostic value in multivariate analysis and may serve as a robust preoperative tool in resectable PDAC. The CALLY index, although not independently significant in multivariate analysis, showed strong prognostic separation in Kaplan–Meier survival analyses and may still aid in preoperative risk stratification, particularly where access to comprehensive scoring systems is limited.

## 1. Introduction

Pancreatic ductal adenocarcinoma (PDAC) continues to be one of the most aggressive malignancies globally, with only marginal improvements in long-term survival despite advancements in surgical and systemic therapies [1,2]. Although surgical resection followed by adjuvant chemotherapy constitutes the cornerstone of treatment for resectable disease, recurrence rates remain high, primarily due to undetected micrometastases and the biologically aggressive nature of the tumor [3,4]. This underscores the urgent need for tools that can guide individualized treatment strategies by accurately stratifying patients preoperatively [5,6,7]. In recent years, there has been increasing interest in composite inflammatory and nutritional biomarkers as prognostic tools in PDAC [8,9,10,11,12,13,14,15]. Among these, the C-reactive protein-albumin-lymphocyte (CALLY) index and the Heidelberg Prognostic Pancreatic Cancer (HELPP) score have emerged as promising pre-treatment scoring systems. Both scores integrate routinely available laboratory parameters to provide insights into the host-tumor interaction and overall immune-nutritional status [16,17].

The HELPP score, which integrates CRP, albumin, CA19-9, CEA, and platelet count, has been demonstrated to effectively stratify overall survival across diverse clinical contexts, including both resectable and unresectable cases, surpassing traditional biomarkers such as CA19-9 [16,18]. Platelets contribute to cancer progression by providing a pro-coagulant surface, promoting tumor-cell protection and dissemination, and releasing angiogenic and inflammatory mediators [19]. Similarly, the CALLY index has shown predictive value for both recurrence and survival outcomes in patients undergoing surgical resection [20].

Concurrently, recent developments in treatment paradigms underscore the significance of systemic approaches even in the early stages of PDAC. Adjuvant chemotherapy has become a standard practice, with multi-agent regimens such as mFOLFIRINOX demonstrating survival benefits over gemcitabine alone [21]. Nevertheless, its efficacy is not consistent across all patient subgroups, underscoring the necessity for preoperative prognostic stratification. Recent guidelines also emphasize the importance of incorporating biological factors—not just anatomical criteria—into resectability assessments [3,4,5,21]. In parallel, neoadjuvant treatment strategies, particularly for borderline resectable or biologically aggressive tumors, have gained increasing attention in contemporary treatment guidelines [22].

This study seeks to assess and compare the prognostic accuracy of the HELPP score and the CALLY index in patients with resectable PDAC who have undergone curative-intent surgery followed by adjuvant chemotherapy. We hypothesize that these scoring systems can effectively identify patients at elevated risk of early recurrence and diminished survival.

## 2. Materials and Methods

### 2.1. Study Design

This retrospective, single-center cohort study was conducted to assess and compare the prognostic significance of two systemic inflammation-based markers—the C-reactive protein-albumin-lymphocyte (CALLY) index and the HELPP score—in patients with non-metastatic PDAC who underwent curative-intent surgery followed by adjuvant therapy.

### 2.2. Patient Selection

Patients with histopathologically confirmed non-metastatic PDAC who underwent curative-intent surgical resection between January 2017 and June 2025 at the Department of Medical Oncology, Faculty of Medicine, Kocaeli University, were retrospectively evaluated, provided they had a minimum follow-up period of six months.

All surgical candidates underwent evaluation by a multidisciplinary tumor board, with the decision for curative-intent resection predicated on imaging findings and clinical status. Pancreatic resections were conducted using an open surgical approach. For neoplasms located in the head or neck of the pancreas, a standard pancreaticoduodenectomy (Whipple procedure) was executed, entailing en bloc resection of the pancreatic head, duodenum, gallbladder, and distal stomach. Distal and total pancreatectomies were performed as indicated by the tumor’s location and extent. These procedures are recognized for their association with significant postoperative morbidity; therefore, all patients received standardized perioperative care and follow-up. The surgical stage is defined by the pathological TNM classification established following resection.

The inclusion criteria encompassed patients with a confirmed histopathological diagnosis of PDAC who underwent surgical resection with curative intent and received adjuvant chemotherapy, specifically FOLFIRINOX, gemcitabine-based, or capecitabine-based regimens. Findings from preoperative biopsy or endoscopic ultrasound were not considered in this analysis. Additional criteria required a minimum follow-up period of six months and the availability of comprehensive preoperative laboratory data, including hemoglobin, albumin, C-reactive protein (CRP), lymphocyte count, platelet count, and total protein. Patients were excluded if they presented with metastatic or locally advanced unresectable disease, active infection, chronic inflammatory conditions, or if there was missing laboratory or follow-up information. Patients who had received neoadjuvant chemotherapy were excluded from the study.

Among 122 patients initially screened, 13 were excluded: 10 due to the detection of metastatic disease at or following surgery, and 3 due to incomplete or missing medical records. Thus, a total of 109 patients with a postoperative pathological diagnosis of pancreatic adenocarcinoma were included in the final analysis.

### 2.3. Data Collection and Biomarker Calculation

Clinical, pathological, and treatment-related data were retrospectively extracted from hospital records. Laboratory parameters necessary for score calculations were collected preoperatively, prior to surgical intervention. These parameters were utilized to compute the CALLY index and HELPP score. The CALLY index is defined as: (albumin [g/dL] × lymphocyte count [10^3^/μL])/(CRP [mg/L] × 10^4^) [17,20]. The HELPP score is a composite prognostic index incorporating serum albumin, C-reactive protein (CRP), platelet count, American Society of Anesthesiologists (ASA) score, and tumor marker levels (CEA and CA19-9) [16,18]. The reference ranges were as follows: lymphocyte count (1.05–3.17 × 10^3^/μL), CRP (<5 mg/L), albumin (39.7–49.4 g/L), CEA (0–5.5 μg/L), CA 19-9 (0–39 U/mL), and platelet count (172–380 × 10^9^/L). These ranges reflect the institutional standards of the laboratory at Kocaeli University Hospital at the time of patient evaluation. Tumor diameter was categorized into ≤2 cm, 2–4 cm, and >4 cm, based on previous studies using survival analysis to determine prognostic stratification [18].

### 2.4. Outcome Measures and Statistical Analysis

Follow-up data encompassed recurrence status, disease-free survival (DFS), and overall survival (OS). DFS was defined as the interval from the date of surgery to the initial radiological or clinical indication of tumor recurrence. OS was defined as the period from surgery to the date of death or the last follow-up, whichever occurred first. Survival outcomes were evaluated using Kaplan–Meier survival curves, with comparisons conducted via the log-rank test. Cox proportional hazards models were utilized for both univariate and multivariate analyses to identify independent prognostic factors. CALLY index cut-off values were determined using ROC analysis and Youden’s index, resulting in two groups: low CALLY (≤1.029) and high CALLY (>1.029). The HELPP score was divided into two distinct categories: scores of ≤3 and those exceeding 3. This classification aligns with the methodology of a previously published Chinese study and is corroborated by the survival distribution patterns identified within our dataset. The predictive efficacy of the CALLY index and HELPP scores was evaluated using the ROC curve and AUC analysis. Statistical significance was established at *p* < 0.05. All statistical analyses were conducted using IBM SPSS Statistics for Windows version 29.0 (IBM Corp., Armonk, NY, USA).

## 3. Results

A total of 109 patients were included in the analysis. The median age was 64 years (IQR, 56–70), with 61.5% of patients being under 65 years of age. The sex distribution was 50.5% female and 49.5% male. Diabetes mellitus was present in 33% of the cohort. The majority of tumors were located in the pancreatic head-neck region (73.4%), and most patients underwent pancreaticoduodenectomy (84.4%). Stage II disease was observed in 53.2% of patients. The median tumor size was 2.5 cm (IQR, 0.4–3.5); 47.7% of tumors measured ≤2 cm, 33.9% were >2–4 cm, and 18.3% were >4 cm. Lymphovascular invasion and perineural invasion were noted in 38.5% and 53.2% of cases, respectively. An R1 resection margin was reported in 24.8% of patients. All patients received adjuvant therapy, with 78.9% receiving chemotherapy alone and 21.1% receiving chemoradiotherapy. The most commonly administered chemotherapy regimen was FOLFIRINOX (48.6%), followed by gemcitabine monotherapy (34.9%), FOLFOX (11.0%), and capecitabine (5.5%). Median serum CEA and CA19-9 levels were 2.6 µg/L and 105.1 U/mL, respectively. Median CRP, albumin, platelet, and lymphocyte values were also reported. A low CALLY score (≤1.029) was observed in 68.8% of patients, while 43.1% had high HELPP scores (>3). These descriptive characteristics are summarized in Table 1.

Pairwise log-rank analysis demonstrated significant survival differences among the HELPP score categories, as summarized in Table 2. HELPP score 0 showed significant differences with all other categories except score 1 (*p* = 0.013). No significant difference was observed between HELPP scores 1 and 2 (*p* = 0.150); however, score 1 differed significantly from scores 3, 4, 5, and 6 (all *p* < 0.001). Similarly, HELPP scores 3 and 4 did not differ significantly (*p* = 0.744), whereas score 3 showed significant differences when compared with scores 5 (*p* = 0.012) and 6 (*p* < 0.001). Overall, increasing HELPP score was associated with progressively worse survival outcomes.

Although the HELPP study conducted in China does not represent the original Heidelberg development cohort, it provides a practical grouping strategy for survival-based clinical risk stratification. In our cohort, pairwise survival comparisons demonstrated that the major prognostic separation emerged around a score of 3. The absence of a significant difference between HELPP scores 3 and 4 suggests that these categories share a similar risk profile, whereas the significant difference observed between scores 2 and 3 indicates that the transition in risk level occurs at score 3. The HELPP score was evaluated as a binary variable, employing a cut-off of ≤3 versus >3, as elaborated in the Methods section.

The optimal cut-off values for the laboratory parameters were established through ROC curve analysis and Youden’s index. The determined thresholds were as follows: CRP: 14.15 mg/L, albumin: 3.945 g/dL, lymphocyte count: 3.41 × 10^3^/μL, CEA: 2.765 μg/L, and CA19-9: 191.205 U/mL. Based on these thresholds, patients were categorized into high and low groups for each variable. The distribution of these variables according to low and high CALLY and HELPP scores is summarized in Table 3.

ROC curve analysis identified the optimal preoperative CALLY index cut-off value as 1.029 (AUC = 0.869, Sensitivity = 85.4%, Specificity = 81.5%). Consequently, patients were stratified into low CALLY (CALLY ≤ 1.029) and high CALLY (CALLY > 1.029) groups. The low CALLY group comprised 75 patients (68.8%), whereas the high CALLY group included 34 patients (31.2%). Table 3 delineates the clinico-laboratory characteristics of these CALLY groups.

Patients with low CALLY scores were significantly more likely to have elevated CRP (*p* < 0.001), lower albumin (*p* < 0.001), reduced lymphocyte counts (*p* < 0.001), and higher tumor markers (CEA and CA19-9, both *p* < 0.001 and *p* = 0.001, respectively). These patients also had a higher proportion of R1 resections (*p* = 0.000). Similarly, those with HELPP scores > 3 had significantly higher CRP (*p* = 0.012), lower albumin (*p* < 0.001), elevated CEA and CA19-9 levels (both *p* < 0.001), and a 100% rate of R1 resections compared to 56.5% in the HELPP ≤ 3 group (*p* < 0.001). These findings emphasize the prognostic value of both CALLY index and HELPP scores in relation to systemic inflammation, tumor burden, and surgical outcomes. Further details are provided in Table 3.

**Figure 1:**
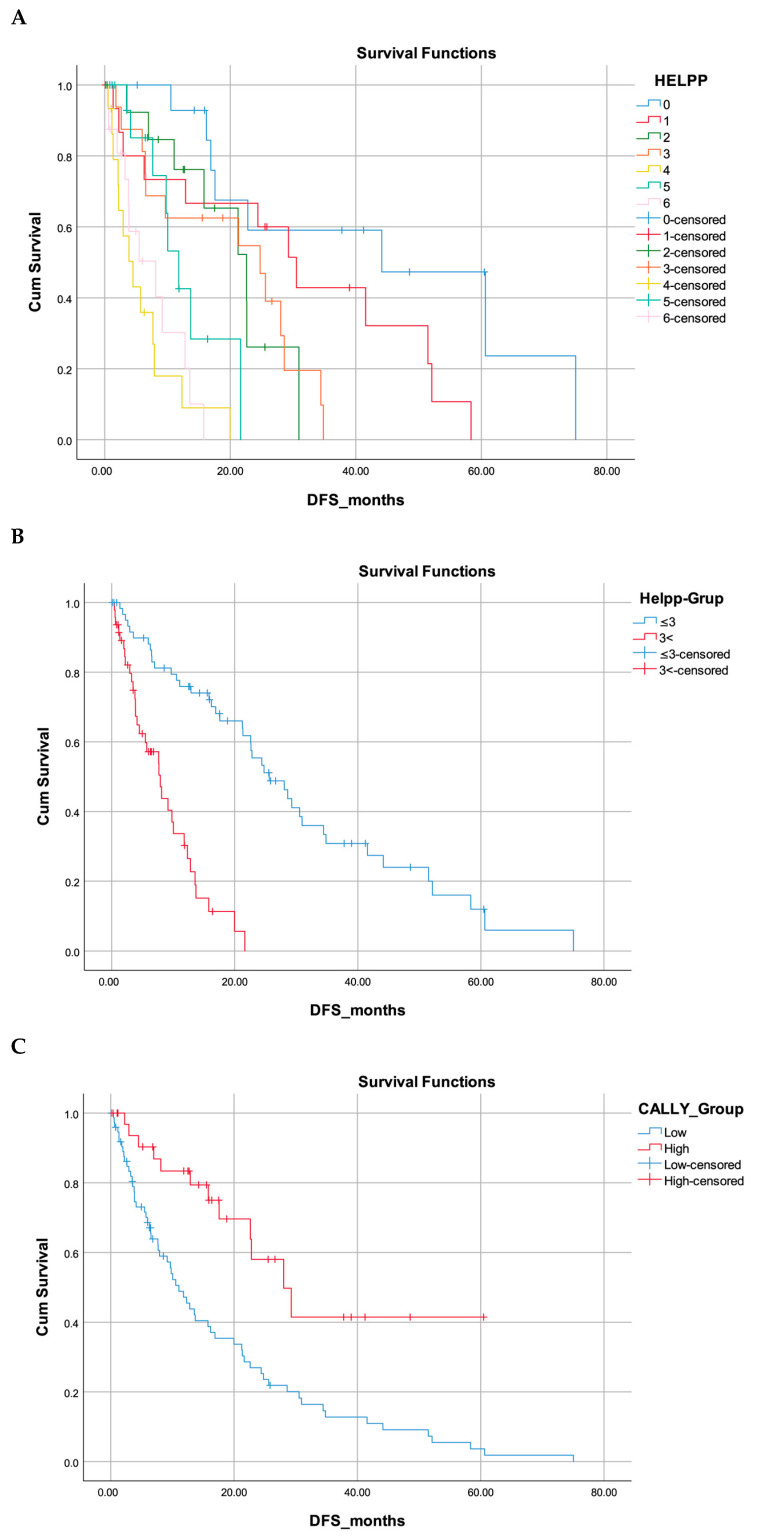
Kaplan–Meier disease-free survival analyses according to HELPP and CALLY scores. (**A**) DFS curves according to HELPP score categories (0–6). DFS decreases progressively with increasing HELPP score (log-rank *p* < 0.001). (**B**) Dichotomized HELPP analysis (≤3 vs. >3). Patients with HELPP > 3 show significantly shorter DFS compared with those with HELPP ≤ 3 (log-rank *p* < 0.001). (**C**) Disease-free survival curves according to the CALLY index (low vs. high). The high-CALLY group exhibits markedly longer DFS compared with the low-CALLY group (log-rank *p* < 0.001).

DFS results are presented in Figure 1. DFS differed significantly across all prognostic scoring systems. The high-CALLY group demonstrated markedly longer DFS compared with the low-CALLY group (median 28.1 vs. 11.1 months; 95% CI 18.9–37.2 vs. 7.8–14.3; log-rank *p* < 0.001). DFS also declined progressively with increasing HELPP scores, showing clear separation between categories (log-rank *p* < 0.001). In the dichotomized analysis, patients with HELPP ≤ 3 had substantially longer DFS than those with HELPP > 3 (median 25.6 vs. 7.9 months; 95% CI 18.7–32.5 vs. 5.0–10.8; log-rank *p* < 0.001).

**Figure 2:**
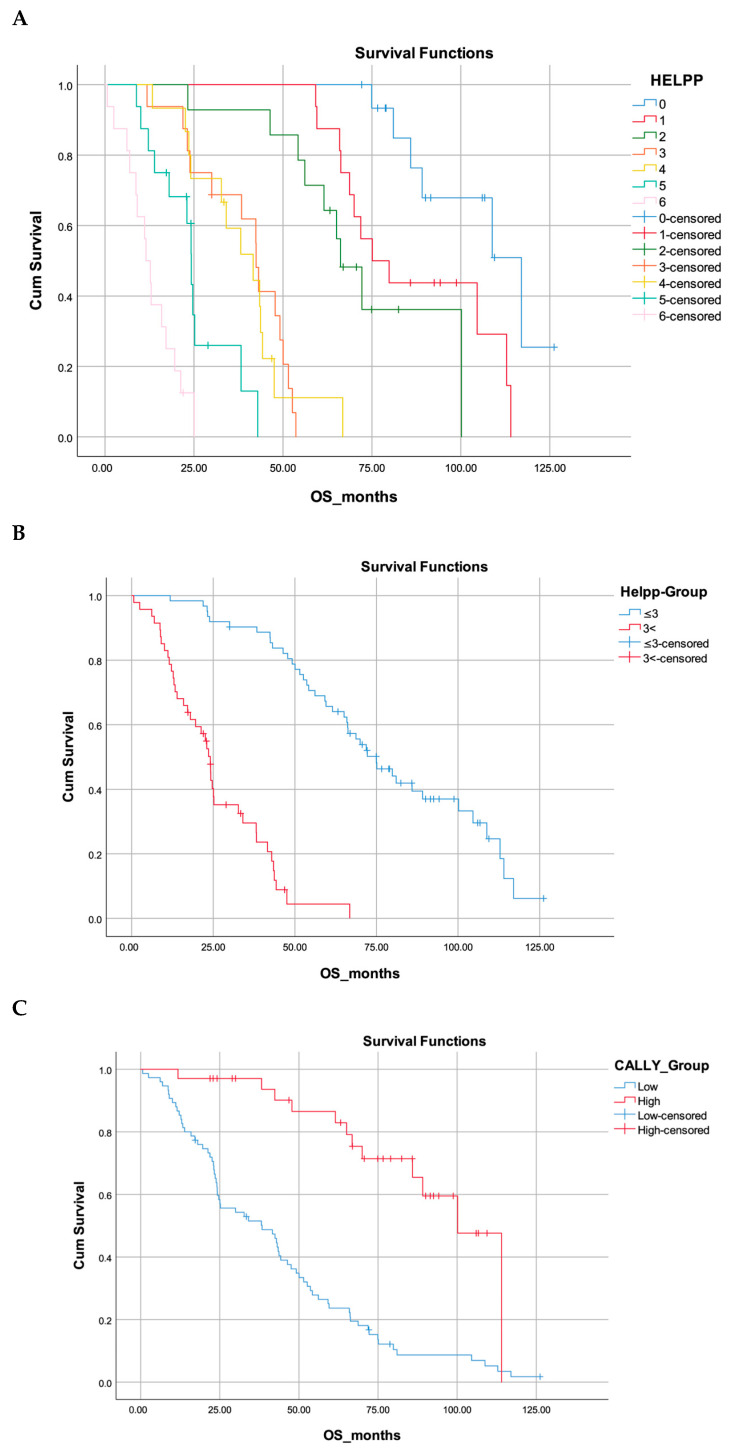
Kaplan–Meier overall survival analyses according to HELPP and CALLY scores. (**A**) Overall survival curves according to HELPP score categories (0–6). A clear decline in survival is observed with increasing HELPP score (log-rank *p* < 0.001). (**B**) Dichotomized HELPP analysis (≤3 vs. >3). Patients with higher HELPP scores show significantly poorer survival compared to the low-score group (log-rank *p* < 0.001). (**C**) Overall survival curves according to the CALLY index (low vs. high). The high-CALLY group demonstrates significantly better survival than the low-CALLY group (log-rank *p* < 0.001).

Figure 2 shows that both the HELPP and CALLY scoring systems provide strong prognostic separation in OS. In the CALLY analysis, the low-CALLY group had a markedly shorter median survival (20.1 months, 95% CI: 20.1–56.7) compared with the high-CALLY group (89.0 months, 95% CI: 89.0–111.3), with a highly significant difference (log-rank *p* < 0.001). Survival also declined progressively across the seven HELPP categories (0–6), with median survival decreasing from 142.9 months in score 0 to 14.7 months in score 6 (log-rank *p* < 0.001). When dichotomized, patients with HELPP ≤ 3 demonstrated substantially longer survival (median 62.5 months, 95% CI: 62.5–87.4) than those with HELPP > 3 (median 21.1 months, 95% CI: 21.1–27.1), again showing a significant difference between risk groups (log-rank *p* < 0.001). Overall, higher HELPP scores and lower CALLY values were consistently associated with reduced survival in Kaplan–Meier analyses.

In the univariate Cox regression analysis, age ≥ 65 (hazard ratio [HR]: 1.890, 95% CI: 1.180–3.030, *p* = 0.006), FOLFOX chemotherapy regimen (HR: 3.867, *p* = 0.021), HELPP score > 3 (HR: 9.960, *p* < 0.001), albumin < 39.4 (HR: 9.099, *p* < 0.001), lymphocyte count > 3.41 (HR: 2.178, *p* = 0.006), CEA ≥ 2.76 (HR: 6.440, *p* < 0.001), CA19-9 ≥ 191.2 (HR: 6.541, *p* < 0.001), low CALLY score (HR: 4.949, *p* < 0.001), and positive surgical margin (HR: 21.449, *p* < 0.001) were significantly associated with overall survival.

In the multivariate analysis, only positive surgical margin (HR: 23.667, *p* < 0.001), HELPP score > 3 (HR: 14.260, *p* < 0.001) remained independently associated with survival. Notably, although the CALLY score showed significance in univariate analysis, it did not retain independent prognostic value in the multivariate model (HR: 3.535, *p* = 0.052). Full results are presented in Table 4.

**Figure 3:**
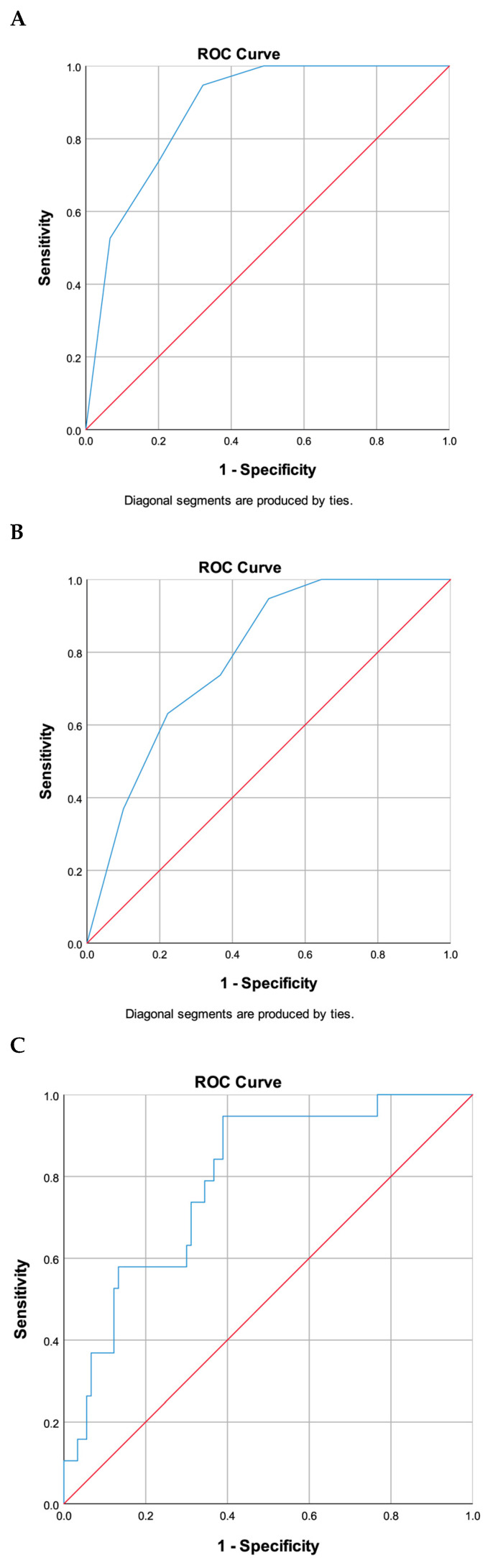

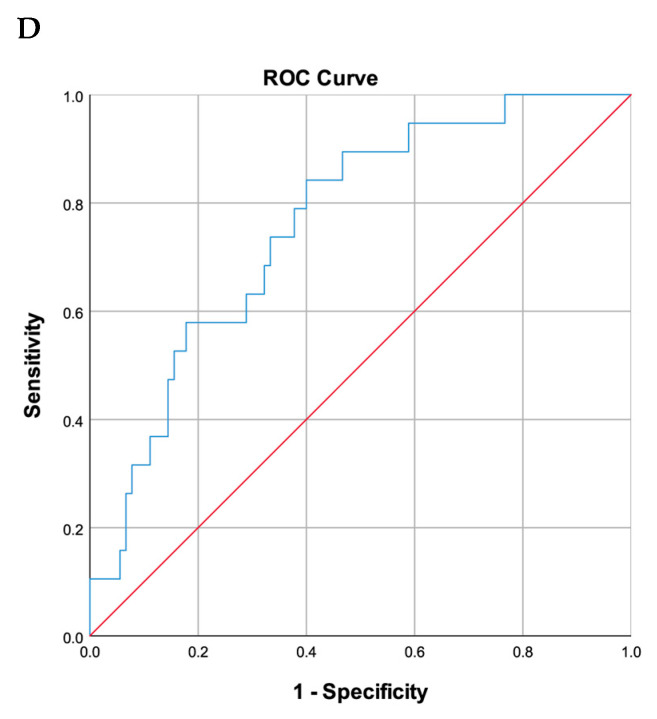
ROC curves demonstrating the prognostic performance of CALLY and HELPP scores for 1-year and 2-year overall survival. The HELPP score demonstrated an AUC of 0.878 (95% CI: 0.809–0.947, *p* < 0.001) at 1 year (**A**) and 0.787 (95% CI: 0.691–0.883, *p* < 0.001) at 2 years (**B**). The CALLY score showed an AUC of 0.761 (95% CI: 0.653–0.869, *p* < 0.001) at 1 year (**C**) and 0.792 (95% CI: 0.691–0.893, *p* < 0.001) at 2 years (**D**). The blue line represents the ROC curve, and the red diagonal line represents the reference line (AUC = 0.5).

The area under the curve (AUC) values for the HELPP score were 0.878 (95% CI: 0.809–0.947, *p* < 0.001) for 1-year OS and 0.787 (95% CI: 0.691–0.883, *p* < 0.001) for 2-yearOS. For the CALLY score, the AUC was 0.761 (95% CI: 0.653–0.869, *p* < 0.001) at 1 year and 0.792 (95% CI: 0.691–0.893, *p* < 0.001) at 2 years. While both scores demonstrated significant predictive ability, the HELPP score had superior performance at 1 year compared to the CALLY score. At 2 years, both scores showed comparable predictive performance, with a slightly higher AUC observed for the CALLY score (Figure 3).

## 4. Discussion

In this study, we assessed and compared the prognostic efficacy of two inflammation-based scoring systems—the CALLY index and the HELPP score—within a cohort of 109 patients diagnosed with resectable pancreatic adenocarcinoma who underwent surgical intervention followed by adjuvant therapy. Both scoring systems demonstrated a significant capacity to stratify patients concerning OS and DFS. Patients exhibiting a low CALLY score (≤1.029) or a high HELPP score (>3) experienced notably shorter OS and DFS durations, with both systems showing distinct separation in Kaplan–Meier survival curves (*p* < 0.001 for both comparisons).

Moreover, ROC analyses indicated that the HELPP score exhibited superior predictive accuracy for 1-year survival, with an area under the curve (AUC) of 0.878, in comparison to the CALLY index, which had an AUC of 0.761. However, at the 2-year interval, both scores demonstrated comparable performance, with the CALLY index achieving an AUC of 0.792 and the HELPP score an AUC of 0.787.

In multivariate Cox regression, HELPP > 3 (HR:14.260, *p* < 0.001), R1 resection (HR: 23.667, *p* < 0.001) emerged as independent adverse prognostic factors. While a low CALLY score also correlated with poorer outcomes, it did not reach statistical significance in the multivariate model (HR: 3.535, *p* = 0.052).

Collectively, these findings indicate that the HELPP score may function as a more robust preoperative prognostic tool in resectable PDAC, owing to its independent predictive value in multivariate analysis. Nonetheless, the CALLY index continues to offer significant prognostic stratification in univariate survival analyses and may be regarded as a practical complementary marker, particularly in contexts where simpler inflammation-based tools are preferred.

The CALLY index, initially introduced by Iida et al. in the context of hepatocellular carcinoma, integrates CRP, albumin, and lymphocyte count, thereby reflecting systemic inflammation, nutritional status, and immune function. Its prognostic significance has been validated across various cancer types. Kawahara et al. demonstrated that a higher preoperative CALLY index was significantly associated with improved OS in patients with pancreatic cancer (OS: 22.1 vs. 37.9 months; *p* < 0.001) [9]. Furthermore, studies in gastric cancer (Fukushima et al., *n* = 826) and epithelial ovarian cancer (Wang et al., *n* = 310) have also confirmed its independent predictive value for both OS and DFS, with CALLY thresholds typically ranging between 2 and 3 [11,23]. In our pancreatic cancer cohort, although a CALLY score ≤ 1.029 was significantly correlated with shorter OS and DFS in Kaplan–Meier analysis, it narrowly missed statistical significance in multivariate regression (HR: 3.535, *p* = 0.052). These findings suggest that while the CALLY index may be more limited in complex prognostic models, its consistent performance across cancers and simplicity in clinical application support its utility as a valuable preoperative biomarker.

The HELPP score, developed at Heidelberg University, is predicated on six preoperative parameters: ASA score, CRP, albumin, CA19-9, CEA, and platelet count [16]. The original study demonstrated that the HELPP score predicted survival independently of pathological stage and exhibited superior performance compared to CA19-9 alone. In a subsequent external validation study conducted in China, a HELPP score greater than 3 was similarly associated with poor prognosis and surpassed other inflammatory markers, such as SII, NLR, and GPS, in predictive capability [18]. Within our cohort, a HELPP score exceeding 3 was significantly correlated with a 100% R1 resection rate, elevated inflammatory response, and increased tumor marker levels, and was identified as a robust independent predictor of OS (HR: 1.204, *p* < 0.001).

In our cohort, both scores demonstrated significant correlations with CRP, albumin, CEA, CA19-9, and lymphocyte levels, highlighting their ability to reflect systemic inflammation, nutritional status, and tumor burden [10]. The CALLY index consists solely of routine laboratory markers—CRP, albumin, and lymphocyte count—capturing the interplay between systemic inflammation, nutrition, and immune competence. Our findings are in line with the growing body of literature emphasizing the prognostic significance of biological markers such as CA19-9. Notably, Omiya et al. reported that anatomically resectable pancreatic cancer patients with baseline CA19-9 > 500 U/mL had significantly prolonged survival following intensive neoadjuvant chemotherapy, underscoring CA19-9 as an indicator of occult systemic disease rather than localized tumor burden alone [13]. In contrast, the HELPP score integrates not only laboratory markers (CRP, albumin, platelet count) but also tumor markers (CEA, CA19-9) and the ASA physical status score, thereby offering a broader assessment of both oncologic and clinical status. This multidimensional composition may account for the superior prognostic performance of HELPP, particularly in short-term survival prediction [24].

The findings indicate that both scores are indicative of systemic inflammation, nutritional status, and tumor burden [25]. Importantly, the HELPP score demonstrated stronger correlations with aggressive disease characteristics, suggesting its potential as a more comprehensive tool for preoperative risk stratification.

From a clinical standpoint, the CALLY index is notably accessible due to its reliance on routinely available parameters, rendering it particularly advantageous in resource-constrained environments. Conversely, the HELPP score, despite its complexity, offers a more detailed and robust prognostic assessment by incorporating a range of inflammatory and tumor-related markers. This characteristic enhances its potential utility in high-volume centers where comprehensive preoperative evaluations are feasible.

Both scores offer potentially valuable prognostic insights that may enhance the personalization of postoperative management strategies. Patients exhibiting elevated HELPP scores might warrant closer surveillance, whereas those with lower scores could potentially avoid unnecessary interventions. However, the incorporation of these inflammation-based scoring systems into clinical practice will be contingent upon the outcomes of future prospective studies. It is important to note that the current study was specifically designed to assess the prognostic predictive value of the HELPP score and CALLY index, rather than to inform therapeutic decision-making.

This study has several limitations. The strengths of this study include its homogeneous patient population and single-center design, which ensure data consistency. However, the retrospective nature and limited sample size of the study necessitate further validation. As the HELPP score is relatively new in the literature, broader prospective and multicenter studies are required to confirm its utility across diverse patient populations.

## 5. Conclusions

Among the two scoring systems evaluated, the HELPP score exhibited independent prognostic value in multivariate analysis and may serve as a robust preoperative tool for resectable pancreatic cancer. Although the CALLY index did not achieve statistical significance in the multivariate model, it demonstrated clear prognostic separation in univariate survival analysis. Given its simplicity and reliance on routine laboratory markers, the CALLY index may still hold potential as a supportive risk stratification tool, particularly in resource-limited settings. The findings further substantiate that the integration of inflammation-based and biochemical markers into preoperative prognostic models facilitates a more nuanced and clinically significant risk stratification, surpassing traditional clinicopathological factors.

## Figures and Tables

**Table 1:** Baseline demographic and clinical characteristics of patients (*n* = 109). Continuous variables are presented as median (interquartile range, IQR).

Characteristic	Value (*n* = 109)
Age (years)	
Median (IQR)	62 (21–82)
<65	67 (61.5%)
≥65	42 (38.5%)
Sex	
Female	55 (50.5%)
Male	54 (49.5%)
Diabetes Mellitus	
Absent	73 (67.0%)
Present	36 (33.0%)
Tumor location	
Head-neck	80 (73.4%)
Body	17 (15.6%)
Tail	12 (11.0%)
Surgical procedure	
Pancreaticoduodenectomy (PD)	92 (84.4%)
Distal pancreatectomy	15 (13.8%)
Total pancreatectomy	2 (1.8%)
ASA Score	
1	32 (29.4%)
2	35 (32.1%)
3	42 (38.5%)
TNM classification (pathological)	
I	33 (30.3%)
II	58 (53.2%)
III	18 (16.5%)
Tumor size (cm), median (IQR)	2.5 (0.4–3.5)
Tumor size category	
≤2 cm	52 (47.7%)
2–4 cm	37 (33.9%)
>4 cm	20 (18.3%)
LVI	
Absent	67 (61.5%)
Present	42 (38.5%)
PNI	
Absent	51 (46.8%)
Present	58 (53.2%)
Resection Margin	
R0	82 (75.2%)
R1	27 (24.8%)
Adjuvant Therapy Type	
Chemotherapy alone	86 (78.9%)
Chemoradiotherapy	23 (21.1%)
Chemotherapy Regimen	
FOLFIRINOX	53 (48.6%)
Gemcitabine monotherapy	38 (34.9%)
Capecitabine monotherapy	6 (5.5%)
FOLFOX	12 (11.0%)
Laboratory Parameters), median (IQR)	
CEA (µg/L)	2.66 (0.5–6)
CA19-9 (U/mL)	105.1 (5–391.5)
CRP (mg/L)	15.2 (8.9–19.8)
Albumin (g/L)	39.6 (34–45)
Platelet (×10^9^/L)	286 (155–491)
Lymphocyte (10^3^/μL)	3.44 (1.86–5.9)
CALLY Score	
Low (≤1.029)	75 (68.8%)
High(>1.029)	34 (31.2%)
HELPP Score	
≤3	62 (56.9%)
>3	47 (43.1%)

Abbreviations: ASA, American Society of Anesthesiologists; CEA, carcinoembryonic antigen; CA19-9, carbohydrate antigen 19-9; CRP, C-reactive protein; IQR, interquartile range; LVI, lymphovascular invasion; PNI, perineural invasion; R0/R1, resection margin status (R0 = negative; R1 = microscopic positive); PD, pancreaticoduodenectomy; SD, standard deviation; HELPP, Heidelberg Prognostic Pancreatic Cancer Score; CALLY, C-reactive protein–albumin–lymphocyte index.

**Table 2:** Pairwise Log-Rank (Mantel–Cox) Comparisons of HELPP Categories.

HELPP	0	1	2	3	4	5	6
0	—	χ^2^ = 6.224, *p* = 0.013	χ^2^ = 12.467, *p* < 0.001	χ^2^ = 35.401, *p* < 0.001	χ^2^ = 30.486, *p* < 0.001	χ^2^ = 27.977, *p* < 0.001	χ^2^ = 31.854, *p* < 0.001
1	χ^2^ = 6.224, *p* = 0.013	—	χ^2^ = 2.069, *p* = 0.150	χ^2^ = 35.401, *p* < 0.001	χ^2^ = 26.019, *p* < 0.001	χ^2^ = 27.977, *p* < 0.001	χ^2^ = 31.854, *p* < 0.001
2	χ^2^ = 12.467, *p* < 0.001	χ^2^ = 2.069, *p* = 0.150	—	χ^2^ = 21.301, *p* < 0.001	χ^2^ = 14.002, *p* < 0.001	χ^2^ = 20.073, *p* = 0.004	χ^2^ = 27.304, *p* < 0.001
3	χ^2^ = 35.401, *p* < 0.001	χ^2^ = 35.401, *p* < 0.001	χ^2^ = 21.301, *p* < 0.001	—	χ^2^ = 0.107, *p* = 0.744	χ^2^ = 8.157, *p* = 0.012	χ^2^ = 23.798, *p* < 0.001
4	χ^2^ = 30.486, *p* < 0.001	χ^2^ = 26.019, *p* < 0.001	χ^2^ = 14.002, *p* < 0.001	χ^2^ = 0.107, *p* = 0.744	—	χ^2^ = 6.382, *p* = 0.012	χ^2^ = 23.360, *p* < 0.001
5	χ^2^ = 27.977, *p* < 0.001	χ^2^ = 27.977, *p* < 0.001	χ^2^ = 20.073, *p* = 0.004	χ^2^ = 8.157, *p* = 0.012	χ^2^ = 6.382, *p* = 0.012	—	χ^2^ = 10.049, *p* = 0.002
6	χ^2^ = 31.854, *p* < 0.001	χ^2^ = 31.854, *p* < 0.001	χ^2^ = 27.304, *p* < 0.001	χ^2^ = 23.798, *p* < 0.001	χ^2^ = 23.360, *p* < 0.001	χ^2^ = 10.049, *p* = 0.002	—

Abbreviations: HELPP = Heidelberg Prognostic Pancreatic Cancer Score; χ^2^ = chi-square; *p* = *p*-Value.

**Table 3:** Biochemical and Clinical Variables Compared Between CALLY and HELPP Groups.

Category (*n* [%] of Total, *N* = 109)	CALLY Low (*n* = 75, 68.8%)	CALLY High (*n* = 34, 31.2%)	*p* (CALLY)	HELPP ≤ 3 (*n* = 62, 56.9%)	HELPP > 3 (*n* = 47, 43.1%)	*p* (HELPP)
Sex			0.160			0.336
Female	42 (56.0%)	13 (38.2%)		28 (45.2%)	27 (57.4%)	
Male	33 (44.0%)	21 (61.8%)		34 (54.8%)	20 (42.6%)	
Age			0.098			0.102
<65	50 (66.7%)	17 (50.0%)		28 (45.2%)	14 (29.8%)	
≥65	25 (33.3%)	17 (50.0%)		34 (54.8%)	33 (70.2%)	
BMI			0.343			0.770
<18.5	5 (6.7%)	2 (5.9%)		4 (6.5%)	3 (6.4%)	
18.5–22.9	20 (26.7%)	14 (41.2%)		19 (30.6%)	15 (31.9%)	
23–24.9	21 (28.0%)	5 (14.7%)		17 (27.4%)	9 (19.1%)	
≥25	29 (38.7%)	13 (38.2%)		22 (35.5%)	20 (42.6%)	
Diabetes Mellitus			0.223			0.844
No	53 (70.7%)	20 (58.8%)		42 (67.7%)	31 (66.0%)	
Yes	22 (29.3%)	14 (41.2%)		20 (32.3%)	16 (34.0%)	
Tumor Location			0.335			0.529
Head/Neck	52 (69.3%)	28 (82.4%)		47 (75.8%)	33 (70.2%)	
Body	13 (17.3%)	4 (11.8%)		10 (16.1%)	7 (14.9%)	
Tail	10 (13.3%)	2 (5.9%)		5 (8.1%)	7 (14.9%)	
TNM classification (pathological)			0.433			0.990
1	21 (28.0%)	12 (35.3%)		19 (30.6%)	14 (29.8%)	
2	43 (57.3%)	15 (44.1%)		33 (53.2%)	25 (53.2%)	
3	11 (14.7%)	7 (20.6%)		10 (16.1%)	8 (17.0%)	
Surgical procedure			0.306			0.670
Pancreaticoduodenectomy (PD)	66 (88.0%)	26 (76.5%)		54 (87.1%)	38 (80.9%)	
Distal pancreatectomy	8 (10.7%)	7 (20.6%)		7 (11.3%)	8 (17.0%)	
Total pancreatectomy	1 (1.3%)	1 (2.9%)		1 (1.6%)	1 (2.1%)	
Adjuvant Therapy			0.355			0.969
Chemotherapy Only	61 (81.3%)	25 (73.5%)		49 (79.0%)	37 (78.7%)	
RT + CT	14 (18.7%)	9 (26.5%)		13 (21.0%)	10 (21.3%)	
Received Chemotherapy Regimen			0.068			0.086
FOLFIRINOX	42 (56.0%)	11 (32.4%)		24 (38.7%)	29 (61.7%)	
Gemcitabine	21 (28.0%)	17 (50.0%)		27 (43.5%)	11 (23.4%)	
Capecitabine	5 (6.7%)	1 (2.9%)		3 (4.8%)	3 (6.4%)	
FOLFOX	7 (9.3%)	5 (14.7%)		8 (12.9%)	4 (8.5%)	
Histological grade			0.212			0.269
Well differentiated	21 (28.0%)	13 (38.2%)		21 (33.9%)	13 (27.7%)	
Moderately differentiated	26 (34.7%)	14 (41.2%)		25 (40.3%)	15 (31.9%)	
Poorly differentiated	28 (37.3%)	7 (20.6%)		16 (25.8%)	19 (40.4%)	
LVI			0.640			0.402
Absent	45 (60.0%)	22 (64.7%)		36 (58.1%)	31 (66.0%)	
Present	30 (40.0%)	12 (35.3%)		26 (41.9%)	16 (34.0%)	
PNI			0.651			0.701
Absent	34 (45.3%)	17 (50.0%)		30 (48.4%)	21 (44.7%)	
Present	41 (54.7%)	17 (50.0%)		32 (51.6%)	26 (55.3%)	
Surgical Margin			0.000			<0.001
R0	64 (85.3%)	18 (52.9%)		35 (56.5%)	0 (0.0%)	
R1	11 (14.7%)	16 (47.1%)		27 (43.5%)	47 (100.0%)	
ASA Score			0.722			0.895
1	23 (30.7%)	9 (26.5%)		18 (29.0%)	14 (29.8%)	
2	25 (33.3%)	10 (29.4%)		21 (33.9%)	14 (29.8%)	
3	27 (36.0%)	15 (44.1%)		23 (37.1%)	19 (40.4%)	
CRP			0.000			0.072
<14.15	12 (16.0%)	31 (91.2%)		29 (46.8%)	14 (29.8%)	
≥14.15	63 (84.0%)	3 (8.8%)		33 (53.2%)	33 (70.2%)	
Albumin			0.001			<0.001
≤39.4	33 (44.0%)	4 (11.8%)		0 (0.0%)	37 (78.7%)	
>39.4	42 (56.0%)	30 (88.2%)		62 (100.0%)	10 (21.3%)	
Lymphocyte			0.000			0.978
≤3.41	66 (88.0%)	13 (38.2%)		45 (72.6%)	34 (72.3%)	
>3.41	9 (12.0%)	21 (61.8%)		17 (27.4%)	13 (27.7%)	
CEA			0.000			<0.001
<2.76	29 (38.7%)	27 (79.4%)		55 (88.7%)	1 (2.1%)	
≥2.76	46 (61.3%)	7 (20.6%)		7 (11.3%)	46 (97.9%)	
CA19-9			0.001			<0.001
<191.2	50 (66.7%)	33 (97.1%)		62 (100.0%)	21 (44.7%)	
≥191.2	25 (33.3%)	1 (2.9%)		0 (0.0%)	26 (55.3%)	
Tumor Size (pathological)			0.599			0.216
≤2 cm	36 (48.0%)	16 (47.1%)		28 (45.2%)	24 (51.1%)	
2–4 cm	27 (36.0%)	10 (29.4%)		25 (40.3%)	12 (25.5%)	
>4 cm	12 (16.0%)	8 (23.5%)		9 (14.5%)	11 (23.4%)	

Abbreviations: CRP, C-reactive protein; Alb, albumin; CEA, carcinoembryonic antigen; CA19-9, cancer antigen 19-9; LVI, lymphovascular invasion; PNI, perineural invasion; ASA, American Society of Anesthesiologists; R0/R1, negative/positive resection margins; IQR, interquartile range; KT, chemotherapy; BMI, body mass index.

**Table 4:** Cox Proportional Hazards Analysis for Overall Survival (OS).

Variable	Univariate HR (95% CI)	*p*	Multivariate HR (95% CI)	*p*
Sex		0.222		0.056
Female	Reference		Reference	
Male	0.692 (0.442–1.083)		2.393 (1.158–4.946)	
Age (years)		0.006		0.888
<65	Reference		Reference	
≥65	1.890 (1.180–3.030)		1.049 (0.537–2.050)	
BMI (kg/m^2^)		0.774		0.478
<18.5	Reference		Reference	
18.5–22.9	1.090 (0.419–2.837)	0.859	0.600 (0.164–2.197)	0.440
23–24.9	0.963 (0.566–1.639)	0.890	0.760 (0.353–1.638)	0.484
≥25	1.294 (0.728–2.301)	0.381	1.328 (0.580–3.041)	0.502
Diabetes		0.294		0.482
No	Reference		Reference	
Yes	1.287 (0.802–2.066)	0.296	0.789 (0.407–1.528)	
Tumor location		0.271		0.436
Head/Neck	Reference		Reference	
Body	0.598 (0.303–1.179)	0.138	0.577 (0.171–1.949)	0.376
Tail	0.765 (0.339–1.726)	0.519	0.351 (0.069–1.792)	0.208
TNM classification (pathological)		0.082		0.005
Stage I	Reference		Reference	
Stage II	2.219 (1.074–4.589)	0.031	7.539 (2.236–25.413)	0.001
Stage III	1.968 (0.987–3.924)	0.055	3.405 (1.282–9.045)	0.014
Surgical procedure		0.971		0.884
Pankreatoduodenectomy	Reference		Reference	
Distal pancreatectomy	1.139 (0.276–4.700)	0.857	1.444 (0.234–8.898)	0.692
Total pancreatectomy	1.197 (0.262–5.466)	0.817	1.723 (0.194–15.291)	0.625
Adjuvant chemotherapy regimen		0.012		0.192
FOLFORINOX	Reference		Reference	
Gemcitabine	2.806 (1.174–6.707)	0.20	3.243 (0.874–12.027)	0.079
Capecitabine	1.596 (0.656–3.884)	0.303	1.875 (0.569–6.173)	0.301
FOLFOX	3.867 (1.228–12.175)	0.021	5.473 (0.940–31.869)	0.059
Histological grade		0.039		0.495
Well differentiated	Reference		Reference	
Moderately differentiated	0.903(0.525–1.552)	0.711	0.955 (0.443–2.058)	0.907
Poorly differentiated	1.749(1.008–3.032)	0.047	0.650 (0.297–1.424)	0.282
Lymphovascular invasion		0.979		0.121
No	Reference		Reference	
Yes	0.994 (0.639–1.545)	0.979	1.656 (0.875–3.131)	
Perineural invasion		0.772		0.544
No	Reference		Reference	
Yes	0.937 (0.603–1.455)		1.204 (0.661–2.192)	
Surgical margin		<0.001		<0.001
Negative	Reference		Reference	
Positive	21.449 (7.557–60.878)		23.667 (6.068–92.304)	
HELPP score (points)		<0.001		<0.001
≤3	Reference		Reference	
>3	9.960 (5.306–17.940)		14.260 (2.835–71.726)	
ASA score		0.851		0.102
1	Reference		Reference	
2	1.068 (0.620–1.837)	0.813	0.878 (0.419–1.839)	0.729
3	0.909 (0.544–1.521)	0.718	1.139(0.544–2.387)	0.729
CRP		<0.001		0.818
<14.15	Reference		Reference	
≥14.15	3.888 (2.267–6.670)		0.892 (0.338–2.357)	
Albumin		<0.001		0.599
>39.4	Reference		Reference	
≤39.4	9.099 (4.905–16.878)	<0.001	0.756 (0.267–2.143)	
Lymphocyte		0.004		0.390
≤3.41	Reference		Reference	
>3.41	0.459 (0.265–0.796)	0.006	1.466 (0.613–3.507)	
CEA		<0.001		0.215
<2.76	Reference		Reference	
≥2.76	6.440 (3.840–10.790)	<0.001	2.251 (0.625–8.109)	
CA19-9		<0.001		0.667
<191.2	Reference		Reference	
≥191.2	6.541 (3.799–11.263)		1.211 (0.506–2.897)	
CALLY		<0.001		0.052
high	Reference		Reference	
low	4.949 (2.658–9.217)	<0.001	3.535 (0.988–12.651)	
Tumor size (pathological)		0.860		0.658
≤2 cm	Reference		Reference	
2–4 cm	1.171 (0.618–2.218)	0.629	0.850 (0.378–1.912)	0.695
>4 cm	1.193 (0.620–2.293)	0.597	0.675 (0.288–1.583)	0.366

Abbreviations: BMI, body mass index; DM, diabetes mellitus; LVI, lymphovascular invasion; PNI, perineural invasion; ASA, American Society of Anesthesiologists score; CEA, carcinoembryonic antigen; CA19-9, carbohydrate antigen 19-9; CRP, C-reactive protein; HELPP, Heidelberg Prognostic Pancreatic Cancer Score; CALLY, C-reactive protein, albumin, and lymphocyte-based index.

## Data Availability

The datasets generated and/or analyzed during the current study are available from the corresponding author on reasonable request. The data are not publicly available due to ethical restrictions and patient confidentiality.
